# Evaluating Antiplasmodial and Antimalarial Activities of Soybean (*Glycine max*) Seed Extracts on *P. falciparum* Parasite Cultures and *P. berghei*-Infected Mice

**DOI:** 10.1155/2020/7605730

**Published:** 2020-02-17

**Authors:** Kevin Nyandwaro, Job Oyweri, Francis Kimani, Amos Mbugua

**Affiliations:** ^1^Department of Medical Laboratory Science, Jomo Kenyatta University of Agriculture and Technology Nairobi, P.O. Box 90420–80100, Nairobi, Kenya; ^2^Kenya Medical Research Institute, Centre for Biotechnology Research and Development, P.O. Box 54840–0002, Nairobi, Kenya; ^3^Department of Pure and Applied Sciences, Technical University of Mombasa, P.O. Box 90420–80100, Mombasa, Kenya

## Abstract

**Background:**

*Plasmodium* parasite resistance to artemisinin-based combination therapies (ACTs) calls for development of new, affordable, safe, and effective antimalarial drugs. Studies conducted previously on soybean extracts have established that they possess antimicrobial, anti-inflammatory, anticancerous, and antioxidant properties. The activity of such extracts on *Plasmodium* parasite resistance to artemisinin-based combination therapies (ACTs) calls for development of new, affordable, safe, and effective antimalarial drugs. Studies conducted previously on soybean extracts have established that they possess antimicrobial, anti-inflammatory, anticancerous, and antioxidant properties. The activity of such extracts on

**Objectives:**

The aim of this study was to determine the antiplasmodial activity of soybean extracts using *Plasmodium falciparum* cultures, followed by an *in vivo* evaluation of safety and antimalarial activity of the extracts in *Plasmodium berghei* ANKA strain-infected mice.

**Method:**

Aqueous, methanol, and peptide extracts of soybean seeds were prepared. An *in vitro* evaluation of the extracts for antiplasmodial activity was carried out using two *P. falciparum* strains: D6, a chloroquine-sensitive Sierra Leone 1 strain and W2, a chloroquine-resistant Indochina 1 strain. Following the *in vitro* evaluation of the extracts for antiplasmodial activity was carried out using two *in vivo* evaluation of safety and antimalarial activity of the extracts in *P. berghei* ANKA strain. The two extracts were tested for their therapeutic potential (curative test). The peptide extract was further assessed to determine whether it could prevent the establishment of a *P. berghei* ANKA strain. The two extracts were tested for their therapeutic potential (curative test). The peptide extract was further assessed to determine whether it could prevent the establishment of a *P. berghei* ANKA strain. The two extracts were tested for their therapeutic potential (curative test). The peptide extract was further assessed to determine whether it could prevent the establishment of a

**Results:**

Peptide and methanol extracts showed good activity with IC_50_ of 19.97 ± 2.57 *μ*g/ml and 10.14 ± 9.04 *μ*g/ml and 10.14 ± 9.04 *μ*g/ml and 10.14 ± 9.04 *μ*g/ml and 10.14 ± 9.04 *P* < 0.001) in suppression with lower doses.

**Conclusion:**

The results show the presence of antimalarial properties in soybean extracts with higher curative activity when compared to the prophylactic activity. However, more research needs to be conducted on this plant to possibly establish lead compounds.

## 1. Introduction

Globally, malaria cases are approximately 300 million each year with about one million deaths [1]. A majority of the malaria cases and deaths are found in sub-Saharan Africa where mortality is highest among infants and children below 5 years [2]. The use of effective drugs to treat malaria cases is a key intervention. However, antimalarial drug treatments such as artemisinin-based combination therapies (ACTs) face the growing threat of drug resistance [3]. Drug resistance parasites develop following prolonged exposure to antimalarial drugs. This drug pressure facilitates emergence of parasite strains that possess molecular mechanisms that compromise or circumvent drug efficacy. Such is the case with ACT-resistant *P. falciparum* parasites that delay parasite clearance and have been identified in South-East Asia. The growing threat of drug resistance to ACTs and other currently administered drugs necessitates the development of novel products for malaria treatment [4].

About 122 drugs have been produced from 94 species of plants through discovery of ethno botanical leads [5]. A majority of traditional medicinal plants are thought to be safe based on the ethno medical knowledge available. Drugs have been generated from plants because of their availability, efficacy, and their mode of action [6]. Nonetheless, more studies are necessary to discover, evaluate, and validate new drugs from plants sources [7].

Soybeans (*Glycine max*) belong to the large botanical family *Leguminosae*, and they grow in tropical, subtropical, and temperate climatic regions. Their seeds measure 8 to 10 mm in diameter and grow within a pod similar to that of peas [8]. They contain 20% oil content and are rich in phytochemicals which have health benefits, hence making them neutraceuticals or functional food crops. [9]. Traditionally, *Glycine max* leaves, roots, and seeds are used for treatment of wide range of ailments including malaria [10]. The plant has been demonstrated to possess significant biological activities such as antioxidant [11], anti-inflammatory [12], antidiabetic [13], antidepressants [14], and antimicrobial [15]. Soybeans have a healing potential against atherosclerosis, osteoporosis, and various types of cancers including those affecting the prostate, breast, and uterus [15]. They also contain isoflavones which are thought to be a major contributor to the antioxidative activity of this legume [16]. A previous study on antimalarial properties of soybean fat emulsions showed that *Glycine max* exhibited a promising inhibitory activity against 3D7 strain of *P. falciparum* with IC_50_ values ranging from 8.07 ± 2.13 to 13.02 ± 2.35 *μ*g/ml [17]. The soybean lipid extracts used in this study by Deharo et al. were Intralipid® and Ivelip® which are commercial products used for intravenous lipid supplementation. The study demonstrated the antimalarial potential of soybeans even though radical cure could not be established. The current study set out to assess antiplasmodial and antimalarial activities of soybeans seed extracts besides the lipid extracts. Additionally, a toxicity study was conducted to determine the lethal dose of the extracts.

## 2. Overview of Materials and Methodology of Study Design

The study was a laboratory-based experimental investigation of the potential antiplasmodial and antimalarial activity of soybean (*Glycine max*) extract (SE). The antiplasmodial activity of crude SE was accomplished through evaluation of antiplasmodial activity of crude SE (*in vitro*) on *P. falciparum* cultures and a *Plasmodium berghei* mouse malaria model (*in vivo*) evaluation. The study was carried out at the Centre for Biotechnology Research and development (CBRD) and Animal House in the Kenya Medical Research Institute (KEMRI), respectively.

### 2.1. Sourcing of *Glycine max* Seeds

Soybean seeds variety SB 19 were collected and packed in plastic paper bags from Kenya Agriculture and Livestock Research Organization (KALRO) in Nairobi, Kenya, in October 2018. The seeds were air-dried for 3 weeks at room temperature and pulverized using laboratory mills.

### 2.2. Preparation of Soybean Extracts

Three extracts (water, methanol, and peptide) were prepared from the milled soybean seeds.

#### 2.2.1. Aqueous and Methanol Extract Preparation

100 g of Soybean powder was macerated in 1 litre of respective solvents (water or methanol). After dissolution, the solutions of samples were shaken in an incubator shaker at a moderate temperature of 37°C ± 2 for 7 days. The samples were taken out of shaker afterwards and filtered initially with muslin cloth and then with Whatman filter paper #01 so that a transparent solution was obtained. The aqueous and methanol filtrates were next placed in an open water bath for the solvent to evaporate at 35°C ± 2 for 6-7 hours daily until dried extracts are obtained. The dried extracts were transferred into vials and stored at −20°C for further analysis and future use.

#### 2.2.2. Peptide Extract Preparation

To prepare the peptide extract, 10 g of soybean powder was added to a 100 ml cold extraction buffer (10 mM Na_2_HPO_4_, 15 mM NaH_2_PO_4_, 100 m MKCl, and 1.5% EDTA) at pH 5. The solution was incubated for 3 hours at 4°C. The precipitate was obtained by saturation and centrifuged at 2500 revolutions per minute. The crude peptide pellets were air-dried and stored at −20°C for further analysis and future use. A single extract was prepared for use in both *in vitro* and *in vivo* experiments. Despite prolonged usage of *Glycine max* extract for months, it still demonstrated continued activity, therefore possibling long shelf life.

### 2.3. Preliminary Phytochemical Screening

Screening for phytochemicals of soybean extracts was carried out using standard methods with some modifications [18].

### 2.4. *In Vitro* Culturing of *P. falciparum*

Chloroquine- (CQ-)sensitive (D6) and CQ-resistant (W2) *P. falciparum* strains were used to assess the antiplasmodial activity of soybean extract and fraction of erythrocytic stages *in vitro*. The cultures were maintained at the Centre for Biotechnology Research and Development (CBRD) at the Kenya Medical Research Institute (KEMRI). The *P. falciparum* cultures were maintained according to the method described by Trager and Jensen [18] with minor modifications. *P. falciparum* (D6 and W2) cultures were maintained in fresh human erythrocytes suspended at 4% hematocrit in RPMI 1640 (sigma) containing 0.2% sodium bicarbonate, 0.5% albumax and 45 *μ*g/L hypoxanthine, and 50 *μ*g/L gentamicin and incubated at 37°C under a gas mixture of 5% oxygen, 5% carbon dioxide, and 90% nitrogen. Infected erythrocytes were transferred into fresh complete medium to propagate the culture daily. In *P. falciparum* (INDO strain) culture medium, albumax was replaced by 10% pooled human serum. Parasitemia was determined qualitatively and quantitatively by light microscopy using (Giemsa staining).

### 2.5. *In Vitro* Antiplasmodial Assays Using Soybean Extracts

To assess the antiplasmodial activity of the SE extracts, an *in vitro* serial microdilution assay technique that measures the ability of extracts to inhibit the incorporation of [G3H]-hypoxanthine by *P. falciparum* was used [19]. The experiments were conducted on 96-well plates. First, 25 *μ*l of culture medium was added to all the wells of a 96-well flat-bottomed microtiter plate except those on the 2^nd^ row. 50 *μ*l aliquots of the three extract solutions (aqueous, methanol, and peptide) were added, in duplicate, to the wells of the second row. A Titertek motorized hand diluter (Flow laboratories, Uxbridge UK) was used to make two-fold serial dilution of each soybean extract over a 64-fold concentration range. The stock solutions (100 *μ*g/ml) of the three soybean extracts were diluted two-fold down until a concentration of 1.56 *μ*g/ml was reached. The susceptibility test was performed using an initial parasitemia of 0.4% by adding 200 *μ*l of 1.5% hematocrit *P. falciparum* culture to each well on the 96-well plate, with the exception of the last 4 wells of the first row. Parasitized and nonparasitized erythrocytes were added into all test wells in such a way that 0.4% parasitemia and 1.5% hematocrit was maintained. Before each antiplasmodial test, parasite cultures were synchronized in a ring stage which were obtained following serial treatment with chloroquine. The first row acted as positive control and was therefore not treated. The first four wells served as negative control for normal growth and contained 200 *μ*l of *Plasmodium* blank red blood cells. Chloroquine substituted the extract as reference drugs (positive controls) for standardizing the experiment. After the plates were incubated at 37°C (3% CO_2_, 5% O_2_, and 92% NO_2_) for 48 hrs, radio-labelled hypoxanthine was then added to each well (0.5 *μ*l in 25 *μ*l of the culture medium). The plates were further incubated for 18 hours to allow its uptake by surviving parasites. After the second incubation, the plates were frozen overnight at −20°C to stop the growth. The plates were thawed at room temperature for 1.5 hours before harvesting. Each well was harvested using Betaplate TM cell harvester (Wallace, Zurich Switzerland), which transferred the red blood cells onto a glass fibre filter where they were washed with distilled water. The dried filters were then inserted into a plastic foil with 10 ml of scintillation fluid and counted in a Betaplate TM liquid scintillation counter (Wallac, micro beta Trilux). The results were recorded as counts per minute (cpm) per well at each concentration. Data were transferred to MS Excel software and expressed as a percentage of the untreated controls. The drug concentration capable of inhibiting 50% of the *P. falciparum* (IC_50_) was determined by logarithmic transformation of drug concentration and radioactive counts per minutes (cpm) using the formula:

(1) $$\begin{array}{c} {\text{IC}}_{50}=\text{anti}\log\left[\frac{\log\;X1+\left(\log\;Y_{50}-\log\;Y1\right)\left(\log\;X2-\log\;X1\right)}{\left(\log\;Y2-\log\;Y1\right)}\right], \end{array}$$

where *Y*_50_ was the cpm value midway between parasitized and nonparasitized control cultures, *X*1 and *X*2 were the concentrations, and *Y*1 and *Y*2 were the cpm values, respectively, for the data points above and below the cpm midpoints [20]. The determined SE IC_50_ values are classified in Table 1 as per criteria provided by [21].

### 2.6. Animals Handling, Parasites Inoculation, and Antimalarial Effect of Soybean Extracts

Female Swiss albino mice aging 6–8 weeks old were randomly selected from KEMRI animal facility Nairobi. They were weighed to achieve 20 ± 2 g and housed in well-labeled standard microlon type II cages in the experimental rooms. The relative humidity in the rooms was maintained at 60–70%. Additionally, appropriate ventilation and room temperature were observed. The animals were fed with viable rodent feed and provided with water *ad libitum*. Each cage accommodated five mice per test sample. *Plasmodium berghei* ANKA parasites were the candidates for evaluation of parasite reduction in mice [21]. The parasites were obtained from Kenya Medical Research Institute (KEMRI) Nairobi and maintained by subpassage in mice. Blood infected with *P. berghei* strain ANKA was harvested from donor mice by heart puncture into 15 ml centrifuge tubes containing 1% (w/v) heparin. The parasitized blood was diluted to attain roughly 10^8^ parasitized red blood cells (RBCs) per ml. The experimental animals were intraperitoneally infected with 0.2 ml (2 × 10^7^ parasitized RBCs) and randomly grouped.

### 2.7. Drugs and Administration

The soybean extracts, PBS, and chloroquine were administered orally to the test groups, negative group, and the positive group, respectively. A stainless metallic feeding cannula was used during administration [22].

### 2.8. Assessment of Prophylactic Activity of Soybean Extract

The prophylactic activity of SE and chloroquine were assessed by using the method described by Ryley and Peters [23]. The mice were randomly divided into 5 groups of 5 mice each in a cage. Groups 1 to 3 were orally administered with 200, 400, and 800 mg/kg/day of SE respectively. Group 4 mice were administered with 5 mg/kg/day of chloroquine (positive control), while group 5 mice received a placebo consisting of 3% dimethyl sulfoxide and 10% Tween 80 in PBS (negative control). Administration of the SE continued consecutively for 3 days, i.e., from day 0 to day 3. On the fourth day, the mice were inoculated with *P. berghei* (ANKA). The parasitemia level of each mouse was assessed by blood smear after 72 hours. The survival rate was monitored on a daily basis in all groups for 28 days postinoculation. Giemsa-stained thin blood smears were prepared from the tail of each animal to determine parasitemia and percentage inhibition.

### 2.9. Assessment of Curative Effect of Soybean Extract

The method to evaluate the schizontocidal activity of SE in established infection as described by Tona et al. [24] was used. 0.2 ml of *P. berghei* ANKA (2 × 10^7^parasitized RBCs) was injected intraperitoneally into 25 mice on the first day. Two hours later, the mice were divided randomly into five groups of 5 mice each. The experimental groups were treated orally with a single 0.2 ml dose of SE at one of the three dosage levels of 200, 400, and 800 mg/kg in groups 1 to 3, respectively. For the positive control group (group 4), 5 ml/kg/day of chloroquine was administered. Group 5, which was the negative control, received the placebo (vehicle; 3% dimethyl sulfoxide, 10% tween 80 in PBS). The SE and drugs were administered daily for 3 (days 0 to 3) days. Thin smears of Giemsa were prepared from blood obtained from the tail collected on each day of treatment for the purpose of monitoring the infection. Parasitemia was determined after 4 days (24 hrs after last treatment) by microscopic examination by counting parasites in 4 fields of covering 100 erythrocytes per view of thin blood film sampled from the tail of experiment mouse stained with 10% Giemsa solution. The difference between the mean number of parasites per view in negative control group (100%) and those of experimental groups was calculated and expressed as percentage parasitemia suppression (chemo suppression) for each group, according to Tona et al. [24]:

(2) $$\begin{array}{c} \text{parasitemia suppression}\left(\text{PS}\right)=\left[\frac{\left(A-B\right)}{A}\right]\times 100\text{,} \end{array}$$

where *A* = mean parasitemia in negative control on day 4 and *B* = corresponding parasitemia in the test group.

Percentage parasitemia was calculated as parasitized no. of erythrocytes per 100 erythrocytes, while chemo suppression percentage was taken as inhibition of parasite multiplication relative to the control expressed in percentage. The survival rate was monitored on a daily basis in all groups for 28 days postinoculation. Parasites in the negative control group were assumed to have experienced 1% chemo suppression (potency of the drug to inhibit parasite multiplication).

### 2.10. Determination of Extract Toxicity

The median lethal dose (LD_50_) was determined for estimating acute toxicity of the soybean extract using the method of Lorke [25]. The method involved intraperitoneal administration of different doses (1500, 2500, 3500, and 5000 mg/kg) of SE to the four groups of Swiss albino mice. Afterwards, mortality and manifestation of physical signs in mice for possible toxicity was monitored.

### 2.11. Data and Statistical Analysis

Analyzed data was presented as mean ± standard deviation. *In vitro* tests were performed in duplicate, and data were evaluated by Microsoft Excel 2016 with the aid of nonlinear regression analysis to determine IC_50_. Analysis for the *in vivo* work was achieved using SPSS version 25. Within groups, the statistical significance for contrast of parasitemia reduction and survival time was attained using one-way analysis of variance (ANOVA) and Tukey's Honest Significant Difference post hoc test.

### 2.12. Ethical Consideration

Permission to carry out the study was obtained from Scientific and Ethics Review Unit (SERU), (study SERU no. 3795). The experiments were carried out in compliance with Animal Care and Use Committee (ACUC) Regulations of KEMRI. In addition, the internationally established values for laboratory animal use and care as per WHO recommendations were followed. Intraperitoneal injection was conducted using gauge 23 needles. Death during experimental ends was done in a chamber of carbon (IV) oxide gas followed by incineration.

## 3. Results

### 3.1. Phytochemical Analysis

Eight qualitative chemical tests were performed on the three soybean extracts to determine the presence of phenols, flavonoids, tannins, steroids, alkaloids, saponins, glycosides, and terpenoids. The results showed presence of steroids, alkaloids, and saponins in the aqueous extract and methanol extract (Table 2). Flavanoids, steroids, glycosides, and tannins were present in the peptide extract.

### 3.2. Acute Toxicity Study

The results of acute toxicity showed that the extract caused no mortality within the dose range of 1500–5000 mg/kg in the initial 24 hrs as well as within the successive 14 days. This was a clear indication that doses used were safe. No physical and behavioral signs of overtoxicity such as decreased motor activity, decreased body/limb tone, writhing, respiration, and death amongst others were observed. This proposes that LD_50_ of the extract is greater than 5000 mg/Kg.

### 3.3. *In Vitro* Assessment of Antiplasmodium Activity of SE

The activities of the three different extracts of *Glycine max* against two strains of *P. falciparum* ranged from inactive for the aqueous extract to moderately active for both the methanol and peptide extracts (Table 3). Methanol and peptide extracts showed good activity against D6 and W2 strains. However, methanol extract showed the best activity followed by peptide extract.

Water extract had IC_50_ > 200 *μ*g/ml, thus exhibiting no activity against the two strains of *P. falciparum*.

### 3.4. *In Vivo* Antimalarial Curative Activity of Methanol and Peptide Extracts of *Glycine max*

Two soybean extracts that displayed activity (methanol and peptide) in the *in vitro* studies were next tested for antimalarial activity against *P. berghei* ANKA-infected Swiss albino mice. This was accomplished using the 4-day suppressive method for methanol and peptide extracts. The results are presented in Table 4. Chemosuppresion was established in a dose-dependent manner after four days of antimalarial treatment using SE extracts (200–800 mg/kg). The mean parasitemia in groups treated with methanol ranged from 3.48 ± 0.37 to 5.99 ± 0.18 while that of animals treated with peptide extract varied from 3.61 ± 0.27 to 5.87 ± 0.25. The mean parasitemia in the negative control group was 12.87 ± 0.26. There was a significant percentage parasitemia difference between the test groups when compared with the untreated control group (*P* < 0.001). At 800 mg/kg, the extracts demonstrated the highest chemosuppresion. Remarkably, there was a slight difference in chemosuppresion between the positive control and the two extracts. The extracts were able to prolong survival of the animals after treatment termination in comparison with the negative control.

### 3.5. *In Vivo* Antimalarial Prophylactic Activity of Peptide Extract of *Glycine max*

In prophylactic assay, parasitemia in the negative control group was significantly higher than in any of the test group (*P* < 0.001). All the animals in the positive group displayed suppression of parasitemia of 83.09%. The peptide extracts group suppression of parasitemia was 64.66%, 57.12%, and 43.14% for doses of 800, 400, and 200 mg/kg. The mean parasitemia in groups treated with peptide ranged from 3.94 + 0.46 to 6.35 + 0.22. The results are presented in Table 5. The peptide extract was able to prolong survival of the animals after treatment termination in comparison with the negative control.

## 4. Discussion

Many antimalarial drugs currently available on the market have been developed from plants and natural products [25]. *Plasmodium falciparum* resistance to the existing antimalarials necessitates the development of improved drug interventions [26]. The best solution to this challenge remains to be probably therapeutic plants [27, 28]. Artemisinin derivatives have been used to manage malaria for long [29]. Quinine which is widely used for treatment of malaria was obtained from *Cinchona ofcinalis* plants [30]. Most people in Africa have greatly relied on traditional remedies due to affordability [30]. This study investigated antimalarial activities and safety properties of soybean (*Glycine max*) in order to determine their potential as a source of a novel and cheaper antimalarial agent.

The study clearly demonstrates the potential activity of the *Glycine max* seeds extract against malaria. Phytochemical analysis of methanol and peptide extract revealed the presence of flavanoids, alkaloids, steroids, and glycosides unlike in aqueous extract which had only steroids and alkaloids. It has been reported that oral administration of phytochemicals such as saponins, tannins, and phenols possess ability to suppress cellular immunity [31]. Previous research has shown that the amount of secondary metabolites available in the plants under examination play a greater role in drug research. Formerly, alkaloids, saponins, flavanoids, and tannins have been demonstrated to aid antimicrobial and antimalarial activities of selected medicinal plants [32]. Notably, our results were in conformity with those of Arora [14] who reported presence of alkaloids, flavonoids, tannins, and saponins when determining antimicrobial effects of *Glycine max*. Therefore, the results of this study may have been influenced by single or combination of the mentioned phytochemical ingredients in the crude extracts in exerting activity against malaria.

An ideal antimalarial should be safe without any adverse effects. We ran toxicity study to evaluate the suitability of the use of this plant extract. The outcomes indicated that the concentrations of the extracts applied on the *in vivo* experiments were harmless with no animal death noted within the initial 24 h and successive 14 days at 1500–5000 mg/kg dosage. Importantly, animals stayed alive for the entire four days of the experiments indicating safety as suggested by Satayavivad et al. [33] in their work.

Our findings agree with those of Deharo et al. [17] who carried out the evaluation of antiplasmodial and antimalarial properties of soybean fat emulsions. They recorded antiplasmodial activity of IC_50_ of 13.02 ± 2.35 mg·ml^−1^ upon use of Ivelip test sample indicating quick parasite inhibition. In this study, *in vitro* assays of peptide and methanol extracts showed activity with IC_50_ of 19.97 ± 2.57 *μ*g/ml and 10.14 ± 9.04 *μ*g/ml against D6 strain and 28.61 ± 1.32 *μ*g/ml and 14.87 ± 3.43 *μ*g/ml against W2 strain, respectively. However, we differed on the *in vivo* assay whereby they reported lower percentage parasite suppression upon use of Ivelip test sample, i.e., at 3.2 g·kg^−1^, a reduction of 35 ± 26 was recorded. The difference in the results may be possibly due to difference of the samples used. Nevertheless, their finding and ours provide a clear picture on the potential use of this plant for malaria management. In our study, the *in vivo* assay showed good activity of significant reduction in percentage parasitemia on the test groups compared to the negative control group (*P* < 0.001). *In vivo* antimalarial activity falls into 3 categories of classification: moderate, good, and very good if the extract displayed parasitemia suppression percentage equal to or greater than 50% [34]. In the present study, very good results were obtained in the curative test with methanol and peptide extracts exhibiting over 50% chemosuppresion. Methanol and peptide extracts exhibited high suppressive activity of 72.9% and 71.9% using 800 mg/kg dose, respectively. Notably, there was significant decrease (*P* < 0.001) in activity with lower doses to 64.7% and 64.9% at 400 mg/kg and 53.4% and 54.4% at 200 mg/kg, respectively, in the curative test. A maximum parasite suppression of 72.9% and 71.9% was produced by methanol and peptide in the highest dose of 800 mg/kg and longest survival time compared to other doses. This might be due to the fact that active compounds responsible for the antimalarial activity mostly occur in low levels in natural products and activity may not be detected in lower doses [35]. Likewise, in the prophylactic test, the peptide extract exhibited suppressive activity of 64.7% at 800 mg/kg and 57.1% at 400 mg/kg and 43.1% at 200 mg/kg. Consistent with the above exhibited results, Bonkian et al. [36] used two Sahelian plant extracts that showed a dose-dependent activity. They recorded parasite reduction of 57.5%, 35.9%, and 44.9% at doses of 100, 250, and 500 mg extract/body weight of *Guiera senegalensis* extract, respectively. On the contrary, *Bauhinia rufescens* extract demonstrated parasite suppression activity of 50.6%, 22.2%, and 25.7%. Elsewhere, Menard et al. [37] worked on induction of artemisinin resistance. They were able to isolate a resistant phenotype after 32 successive drug pressure passage cycles. An illustration by Blasco et al. [38] shows that chloroquine was the main stay for malaria eradication after its discovery until parasite resistance undermined its use within a short period calling for an immediate withdrawal. This vividly explains that the use of crude extracts have a chance to delay resistance unlike single compounds. Additionally, crude extracts act as a preliminary step for isolation of pure effective antimalarial agents. Studies conducted previously on *Glycine max* established indeed that it has antioxidant properties [11]. It has been reported that antioxidant activity can inhibit heme polymerization as heme as to be oxidized before polymerization, and the unpolymerized heme is very toxic to malaria [39]. Therefore, this can also be assumed to be one of the factors that lead to presence of antimalarial activity in *Glycine max*. The chemosuppresion data showed that parasite clearance was much more pronounced on the fourth day. This may be attributed to high drug concentration in the blood due to repeated dosing. Interestingly, the parasite reduction activity exhibited on the fourth day suggests that the bioavailability of the chemical components present in the crude extracts is not possibly affected by biotransformation and physiological factors. The mechanism of action of *Glycine max* is not yet known. However, existing literature studies have shown that some plants and seeds exhibit antiplasmodial activity either by causing red blood cells oxidation [40] or by inhibitory protein synthesis [40] depending on their phytochemical constituents. Flavonoids are known to exert antiplasmodial activity by chelating with nucleic acid base pairing of the parasite [36]. Therefore, it is possible that the antiplasmodial activity exhibited by *Glycine max* could have been as a result of the abovementioned ways or by yet a different unknown mechanism. The limitation of the study is that identification of specific compounds responsible for antimalarial activity was not accomplished because this was beyond the scope of the present study. Therefore, we recommend that further analysis should be carried out on *Glycine max* seeds to identify the specific antimalarial compounds present.

## 5. Conclusion

The results of this study provide evidence-based activity of *Glycine max* against the malaria parasite. Therefore, it provides room for future exploitation of the plant in the aforementioned therapeutic effect.

The possibility of *Glycine max* incorporated in food substances should be considered in a wake to determine if the body can develop immune to malaria. The use of *Glycine max* in combination with other herbs for effective treatment [41–48].

## Figures and Tables

**Table 1:** Adopted classification of antiplasmodial activity.

IC_50_ value (*μ*g/ml)	Category of activity
>100	Inactive
50–100	Low
10–50	Moderate
≤10	High

**Table 2:** Results of the phytochemical screening of the three extracts of soybean extract.

Phytochemical	Aqueous extract	Methanol extract	Peptide extract
Phenols	–	–	–
Flavonoids	–	+	+
Tannins	–	–	+
Steroids	+	+	+
Alkaloids	+	+	+
Saponins	+	+	–
Glycosides	–	+	+
Terpenoids	–	–	–

Phytochemicals are indicated as present (+) or absent (–).

**Table 3:** *In vitro* antiplasmodial activity of three extracts of soybeans *Glycine max.*

Treatment	Extract yield (%)	D6–IC_50_ (mean ± SD) (*μ*g/ml)	W2–IC_50_ (mean ± SD) (*μ*g/ml)
Aqueous extract	9.90	>200	>200
Methanol extract	7.40	10.142 ± 9.043	14.867 ± 3.439
Peptide extract	57.50	19.967 ± 2.517	28.613 ± 1.324
Control (CQ)		0.011 ± 3.120	0.091 ± 0.031

**Table 4:** *In vivo* antimalarial activities of crude extract of *Glycine max* in curative test on day 4 and animal survival time.

Treatment	Dose (mg/kg)	Mean ± SD parasitemia (%)	% suppression of parasite	Mean survival time (days)
Methanol extract	200	5.99 ± 0.18	53.45	10.50 ± 0.58
	400	4.54 ± 0.22	64.67	11.25 ± 0.96
	800	3.48 ± 0.37	72.93	16.25 ± 0.96

Peptide extract	200	5.87 ± 0.25	54.39	11.00 ± 0.82
	400	4.52 ± 0.13	64.89	12.40 ± 1.14
	800	3.61 ± 0.27	71.90	15.60 ± 1.52

CQ		1.19 ± 0.36	90.72	28.25 ± 1.50

Vehicle (3% dimethyl sulfoxide and 10% tween 80 in PBS)		12.87 ± 0.26		5.00 ± 0.82

^*∗*^The results are expressed as mean ± SD.

**Table 5:** *In vivo* antimalarial activities of crude extract of *Glycine max* in prophylactic test on day 4 postparasite exposure and animal survival time.

Treatment	Dose (mg/kg)	Mean ± SD parasitemia (%)	% suppression of parasite	Mean survival time (days)
Peptide extract	200	6.35 ± 0.22	43.14	7.50 ± 0.58
	400	4.78 ± 0.30	57.12	9.25 ± 0.96
	800	3.94 ± 0.46	64.66	10.25 ± 0.96

CQ		1.89 ± 0.16	83.09	28.25 ± 1.50

Vehicle (3% dimethyl sulfoxide and 10% Tween 80 in PBS)		11.16 ± 1.15		5.00 ± 0.82

^*∗*^The results are expressed as mean ± SD.

## Data Availability

The data used to support the findings of this study are included within the article.

## Supplementary Materials

Supplementary Materials The supplementary material consists of an excel spread sheet which has all the *in vivo* work data. It includes the summary of different extract doses, negative and positive controls, and how they affected red blood cells in mice. The data were used to determine different parameters used to measure the effect of the extract such as % parasitemia, average parasitemia, chemosuppresion, and mean survival days which are all explained in the article.
