# Supplementary material for: Evaluating Antiplasmodial and Antimalarial Activities of Soybean (Glycine max) Seed Extracts on P. falciparum Parasite Cultures and P. berghei-Infected Mice
Source: J Pathog. 2020 Feb 17;2020:7605730. doi: 10.1155/2020/7605730 (PMC7049415; doi:10.1155/2020/7605730)

Photomicrographs of Giemsa stained blood smears demonstrating parasite manifestation and clearance

Plasmodium infected mice and normal mice

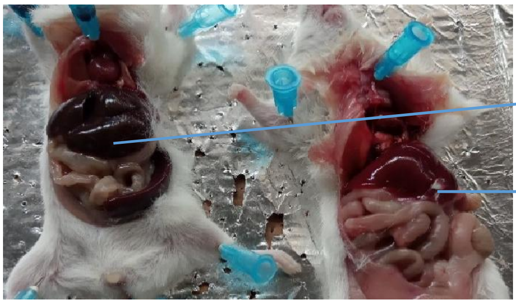

Infected plasmodium liver

Normal liver

Parasitemia in donor mouse

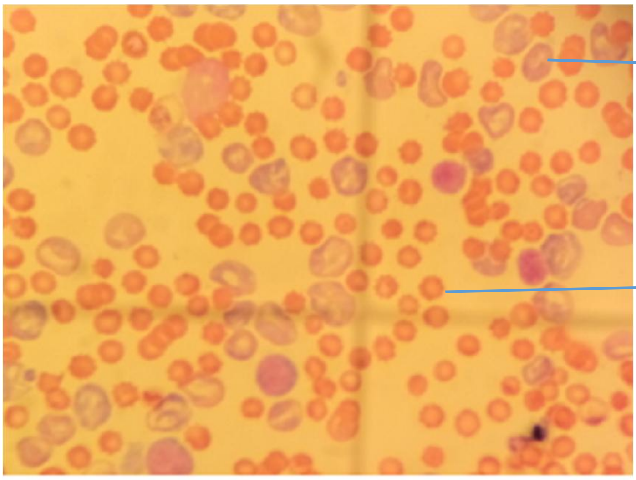

Infected red blood cells

Normal red blood cells

Parasite exposure to peptide extract in mice

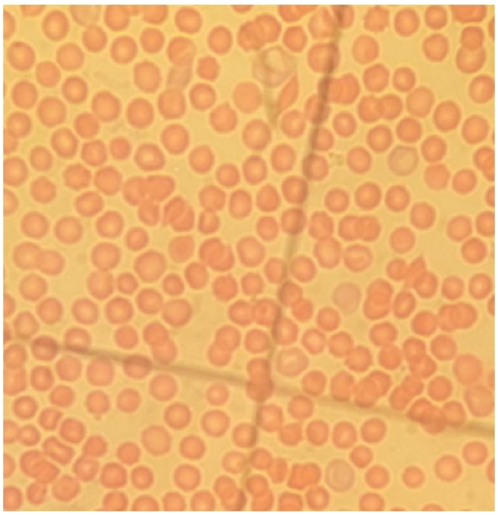

*Parasite exposure to methanol extract in mice

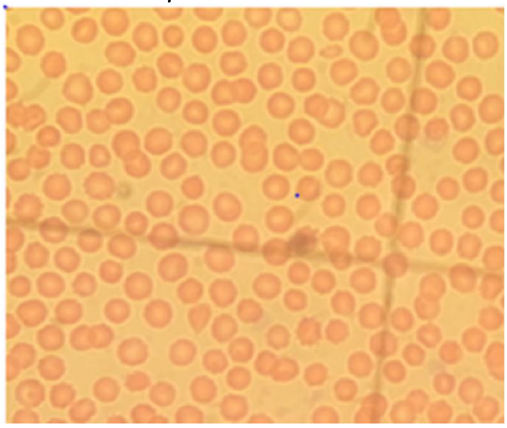

*Parasite exposure to CQ in mice

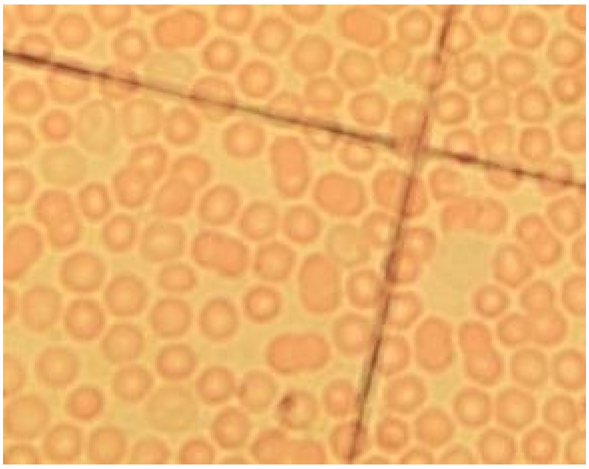

*W2 strain Parasite manifestation after culturing *in vitro*

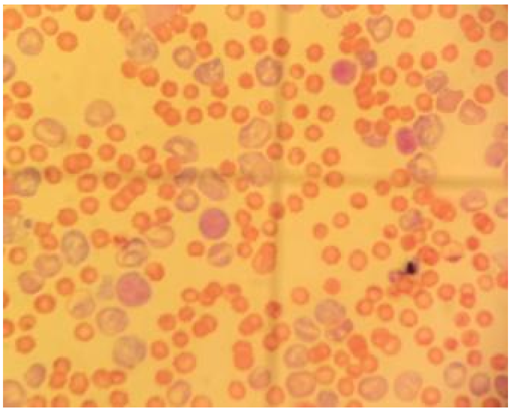

*D6 strain Parasite manifestation after culturing *in vitro*

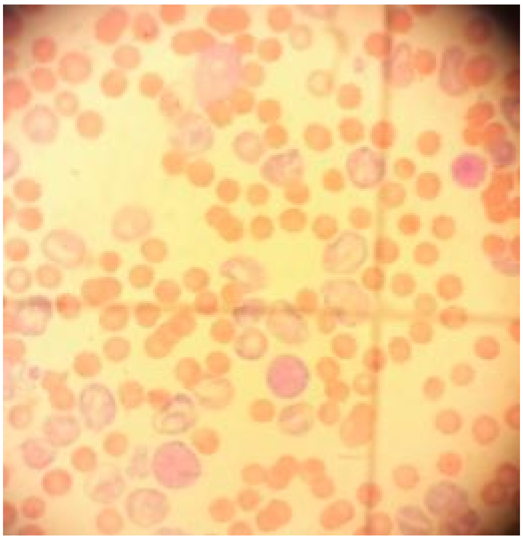

* W2 Parasite inhibition after treatment *in vitro*

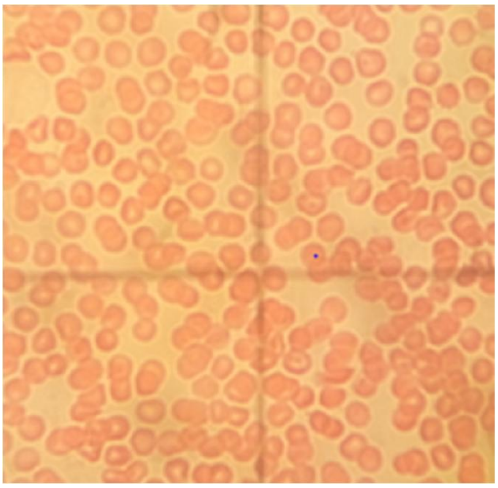

* D6 Parasite inhibition after treatment *in vitro*

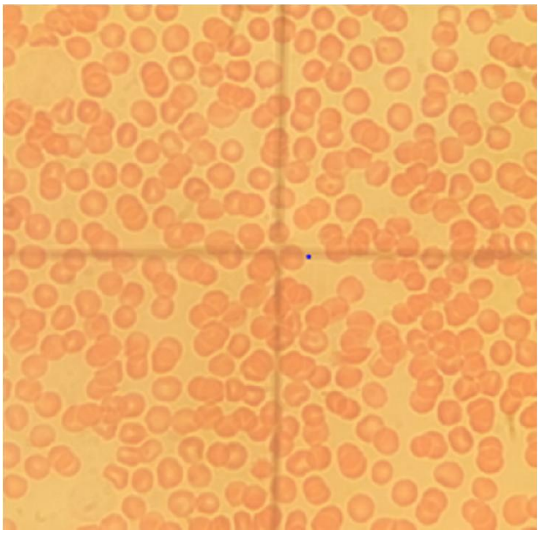

Supplement: Supplementary Materials — The supplementary material consists of an excel spread sheet which has all the in vivo work data. It includes the summary of different extract doses, negative and positive controls, and how they affected red blood cells in mice. The data were used to determine different parameters used to measure the effect of the extract such as % parasitemia, average parasitemia, chemosuppresion, and mean survival days which are all explained in the article. [file 7605730.f1.pdf]
